# 

**Published:** 1861-01

**Authors:** 


### INDEX TO VOL. VIII. OF HALL'S JOURNAL OF HEALTH.

PAGE

Appetite, Instinct of,..............138
Alexander, James W,.............177
A Great Want,...................233
Air and Sunshine,................252
Apples,.........................265

Baths and Bathing,............... 49
Bare Neck and Arms,............. 50
Blackburn, Gideon,.......... .....223
Brag, Thinking,..................264

Coal-Fires,....................... 11
Cities, Health of,...............42, 19
Crying Babies,................... 20
Clerical Reading,................. 22
Cold Feet,..................13, 61, 20
Chambers, Ventilated,............ 41
Colds, How Taken,.............149, 62
Cooling Off Slowly,.............. 64
Consumption, Signs,.............. 66
" Cured,.............. 71
Central Park,.................... 83
Croup,.....................189, 103
Clergyman, Wrecked,.............110
Clock-Flowers, ..................128
Common-Sense,...................167
Choosing a Minister,..............209
Coffee Drinking,..................219
Costiveness, .....................254
Catarrhs,.........................257
Commendatory, ..................267
Cookery and Health,..............285

Death's Doings,................. 12
Dyspepsia,....................... 15
Drinking,........................ 17
Doubtful Witness,................ 25
Dangerous Curiosity,............. 47
Diphtheria,...................... 97
Death's Avenues,................112
" Weapons,..................114
Disease Prevented,...............125
Death-Traps,.....................129
Daughters, Our,..................133
Debts, Pay Your,.............186, 159
Drifting,.........................225
Daring To Do, ...................232
Dollars and Ideas,................236

PAGE

Diet for Invalids,..................259
Damp Feet, (sad case,)............284

Eating,...............122, 246, 44, 16
Exercise, A New,.................130
Eyesight, Failing,.................243
Education,.......................267

Feet,................253, 48, 20, 13
Fireplaces, Open,................ 38
Farm and City Life,.............. 42
Flowers,.........................127
" Faint, yet Pursuing,"............228
Fifteen Follies,...................258
Fruit, Packing,................... 268

Gas,............................ 24
Great Salve,..................... 24
Greed of Gold....................230

Health and Disease,.............. 5
Hair, Beautiful,.................. 19
Healthful Observances,............151
Hungry for Two Years,............165
Human Hopes,....................194
Honesty Rewarded,...............198
Habit, ..........................235
Headache,........................247
Health's Essentials,...............248
Happiest Persons,................262

Ineffable Pills,.................. 25
Instinct of Appetite,..............138

Jacob's Dream,...................191
Jewel in a Blouse,................229

Leaden Water-Pipes, (Vol. 5, 165,).. 8
Life Lost, How,.................. 12
Longevity,................62, 170, 29
Laugh, Pill of,...................115
Late Suppers,.....................121
Literature, Present,...............195
Living to Purpose,................223

Marrying,........... .......268, 107
Marvels of Man,..................131
Mother, Faithful,.................137

| Entry | PAGE |
|---|---|
| Miasm, | 201 |
| Mad Dogs, | 217 |
| Moral Medicine, | 245 |
| Newspapers, | 18 |
| New-York Sights, | 27 |
| Norcom's, Dr., Case, | 71 |
| National Health, | 87 |
| Nelson, Dr. D., | 153 |
| Night Air, | 184 |
| Neuralgia, | 250 |
| Nice Young Man, | 260 |
| Odoriferous Feet, | 13 |
| Open Fireplaces, | 38 |
| Over-Eating, | 44 |
| Old Age, Statistics, | 170, 62 |
| Orchards, | 158 |
| Preventing Disease, | 135, 34 |
| Parks, | 83 |
| Private Things, | 251, 100 |
| Precautions, | 150 |
| Presence of Mind, | 152 |
| Paying Publishers, | 186 |
| Pew System, | 199 |
| Public Speaking, | 202 |
| Pure Air, | 220 |
| Premonitions, | 249 |
| Piano Music, | 267 |
| Pestilence and Plague, | 284 |
| Reading, | 22 |
| Recklessness, | 46 |
| Rheumatism, | 211 |
| Rail-Roading, | 261 |

| Entry | PAGE |
|---|---|
| Sleeping, | 255, 37, 31 |
| Small-Pox, | 32 |
| Smoking Tobacco, | 119, 46 |
| Schools, | 207, 238, 88 |
| Stimulants, | 92 |
| Spring Suggestions, | 94 |
| Suppers, Late, | 121 |
| Skating, | 120 |
| Secret Out, | 122 |
| Science for Christianity, | 132 |
| " Its Achievements, | 141 |
| " Aiding Justice, | 144 |
| Soldier-Health,, | 173, 147 |
| Striking Sentiments, | 192 |
| Sharpe, Ebenezer, | 236 |
| Sick Headache, | 247 |
| Shins, Cracking, | 282 |
| Skating, | 288 |
| Thermometers, | 23 |
| Teeth, | 209, 35 |
| Tobacco, | 183, 46 |
| Throat-Ail, | 51 |
| Trials of Life, | 129 |
| Thinking Well, | 196 |
| The Three P's, | 218 |
| Taking Physic, | 241 |
| Ventilation, | 171, 41 |
| Wonderfully Made, | 86 |
| Wife Search, | 197 |
| Wonderful Woman, | 215 |
| "Wanted," | 224 |
| War, The | 279 |
| Youth Warned, | 223 |

### NOTICE OF WEEKLY NEWSPAPERS.

The following list includes some of the principal newspapers of more than mere local interest, which are regularly received as exchanges. The price and place of publication is of more importance and use to publishers and people than ever so good a "notice." The community forms its estimate of the value of a periodical more from the character of the pieces copied from it than from the commendations of the papers, which commendations are too frequently the result of partialities rather than of convictions, and the public have found this out. We frequently receive letters stating that the writers have for years been wanting to subscribe for "Hall's Journal of Health," but never could learn from their papers the price and place of publication. Hence, to do a real service to our brethren of the press, we make the following list, and will reproduce it from time to time. To our subscribers, one and all, we say, patronize your own local paper first; and if you can spare any more money, then go further from home. "Custom to whom custom is due" is a good general principle. Your local paper brings more or less business to your town or State, and it is your duty to make some return.

**American Presbyterian,** Philadelphia—$2.
**American Railway Times,** Boston—$2.
**American Railway Review,** New-York—$2.
**Banner of the Cross,** Philadelphia, Pa.—Episcopalian—$2.
**Baptist,** Mount Lebanon, La.—$2.
**Banner of the Covenant,** Philadelphia, Pa.—$2—Religious.
**Commercial Advertiser,** Honolula, Sandwich Islands—$2.
**Central Presbyterian,** Richmond, Va.—$2.
**Christian Intelligencer,** New-York—Dutch Reformed—103 Fulton Street.
**Christian Inquirer,** 111 Broadway, New-York—$2—Unitarian.
**Courier and Inquirer,** New-York—$2—Secular—J. Watson Webb, Editor.
**Catholic Herald,** Philadelphia—$2.
**Congregationalist,** Boston—$2.
**Congregational Journal,** Concord, N. H.—$2.
**Christian Advocate,** Baltimore—$2—Methodist.
**Christian Intelligencer,** Richmond, Va.—$2.
**Christian Times,** Chicago, Ill.—$2—Baptist.
**Christian Observer,** Philadelphia—$2—Presbyterian.
**Eagle,** Poughkeepsie, N. Y.—$2.
**Evangelist,** 5 Beekman Street, New-York—$2—Presbyterian.
**Examiner,** New-York—115 Nassau Street—$2—Baptist.
**Evening Post,** Weekly—$2—New-York, corner of Nassau and Liberty Streets.
**Farmers' Advocate,** Chicago, Ill.—$1.50.
**Friend,** Honolula, Sandwich Islands—$1.
**German Reformed Messenger,** Chambersburgh, Pa.—2.
**Home Journal**—107 Fulton Street, New-York—$2—Morris & Willis, Editors.
**Household Journal,** 20 N. William Street—$1.50.
**Herald of Progress,** 274 Canal Street, New-York, A. J. Davis, Editor—Spiritual
**Independent,** 5 Beekman Street, New-York—$2—Congregational.
**Lutheran Standard,** Columbus, O.—$2.
**Lutheran,** Philadelphia, Pa.—$2.
**Lutheran Observer,** Baltimore, Md.—$2.
**Life Illustrated,** 308 Broadway, New-York—$2—Fowler & Wells.
**Michigan Farmer,** Detroit—$2.
**Methodist,** 7 Beekman Street, New-York—$2.
**Miners' Journal,** Pottsville, Pa.—$2.
**Missouri Baptist,** St. Louis—$2.
**New-York Chronicle,** 41 Park Row, New-York—$2—Baptist.
**North-Carolina Presbyterian,** Fayetteville, N. C.—$2.
**New-England Farmer,** Boston, Mass.—$2.
**Olive Branch,** Boston, Mass.—$2.
**Presbyter,** Cincinnati, O.—$2.
**Presbyterian,** Philadelphia, Pa.—$2.50.
**Presbyterian Expositor,** Chicago, Ill.—$2—N. L. Rice, D.D., Editor.
**Presbyterian Herald,** Louisville, Ky.—$2—W. W. Hill, D.D., Editor.
**Presbyterian Banner,** Pittsburgh—$2.
**Pacific,** San Francisco, California—$5—Presbyterian.
**Presbyterian of our Union,** St. Louis, Mo.—$2.
**Religious Telescope,** Dayton, O.—$2.
**Religious Herald,** Richmond, Va.—$2—Baptist.
**Recorder,** Boston, Mass.—$2—Congregational.

